# Supplementary material for: Inherently Area-Selective Atomic Layer Deposition of Manganese Oxide through Electronegativity-Induced Adsorption
Source: Molecules. 2021 May 20;26(10):3056. doi: 10.3390/molecules26103056 (PMC8161048; doi:10.3390/molecules26103056)
Supplement: Supplementary file 1 [file molecules-26-03056-s001.zip › molecules-1203383-supplementary.pdf]

molecules

Supplementary File

Inherently Area-Selective Atomic Layer Deposition of Manganese Oxide through Electronegativity-induced Adsorption

Yi-Cheng Li [1], Kun Cao [1,*], Yu-Xiao Lan [2], Jing-Ming Zhang [2], Miao Gong [1], Yan-Wei Wen [2], Bin Shan [2] and Rong Chen [1,*]

[1] State Key Laboratory of Digital Manufacturing Equipment and Technology, School of Mechanical Science and Engineering, Huazhong University of Science and Technology, 1037 Luoyu Road, Wuhan, Hubei, PR China; yichengli@hust.edu.cn (Y.-C. L.); miaogong@hust.edu.cn (M. G.)

[2] State Key Laboratory of Materials Processing and Die & Mould Technology, School of Materials Science and Engineering, Huazhong University of Science and Technology, 1037 Luoyu Road, Wuhan, Hubei, PR China; M202070873@hust.edu.cn (Y.-X. L.); simonzjm666@gmail.com (J.-M. Z.); ywwen@hust.edu.cn (Y.-W. W.); bshan@mail.hust.edu.cn (B. S.)

* Correspondence: kuncao@mail.hust.edu.cn (K. C.); rongchen@mail.hust.edu (R. C.)

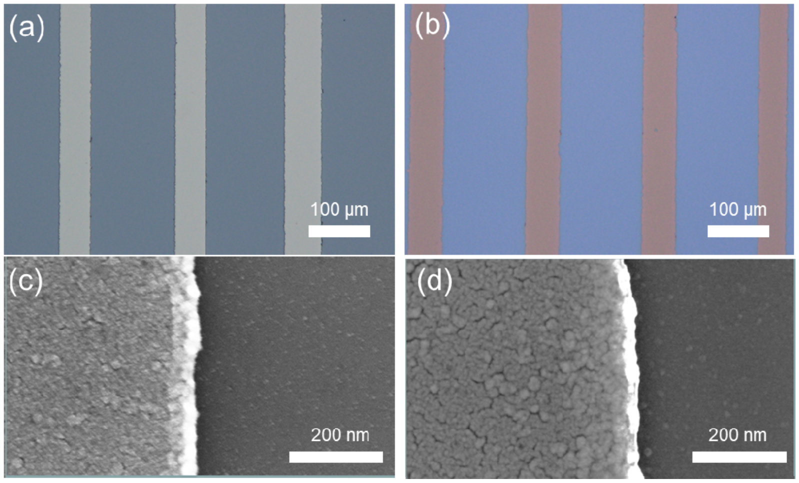

Figure S1. Confocal microscope images of patterned (a) Pt/SiO_2 and (b) Cu/SiO_2 substrate, SEM images of amplified (c) Pt/SiO_2 and (d) Cu/SiO_2 patterns after 50 ALD cycles

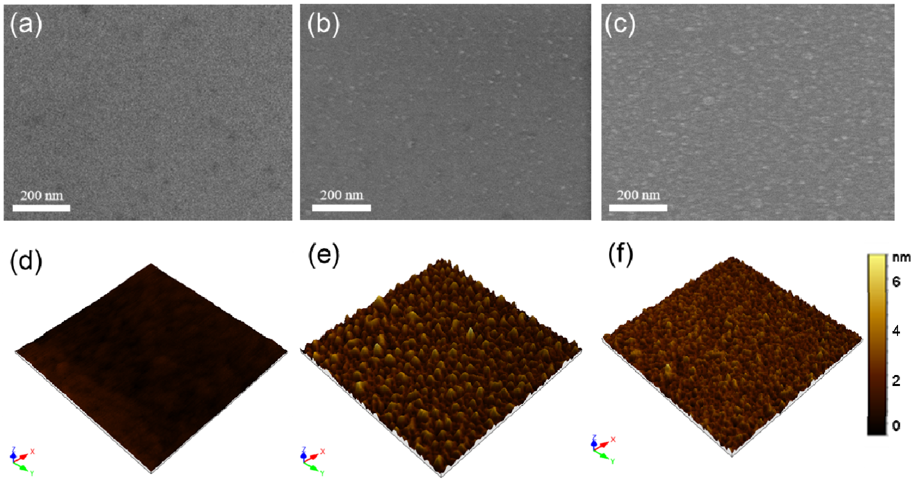

Figure S2. SEM images of (a) bare Si substrate; (b) 50 MnO_x ALD cycles; (c) 400 MnO_x ALD cycles; AFM images of (d) bare Si substrate; (e) 50 MnO_x ALD cycles; (f) 400 MnO_x ALD cycles

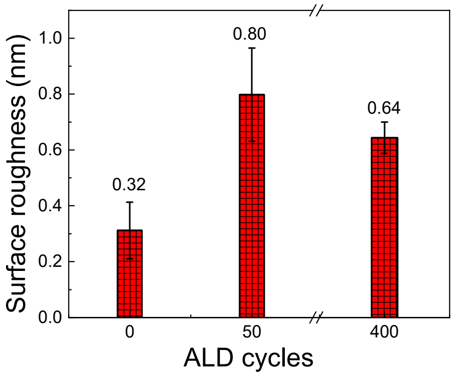

Figure S3. Surface roughness as a function of MnO_x ALD cycles

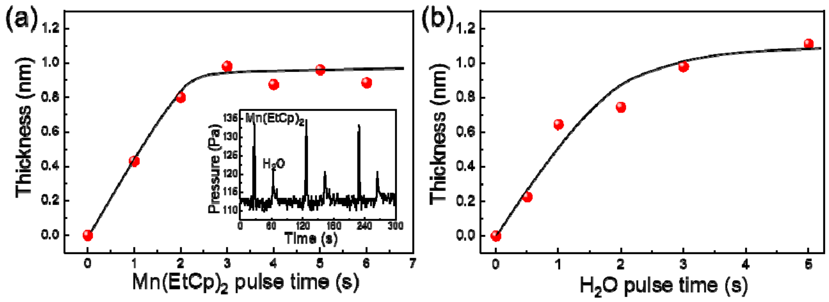

Figure S4. Film thickness with respect to (a) $Mn(EtCp)_2$ pulse time and (b) H_2O pulse time. The inset in (a) is the pressure of dosing $Mn(EtCp)_2$ and H_2O as a function of time. The deposition temperature is 125℃ for (a) and (b).
